# “Programming Is Not That Hard!” When a Science Center Visit Increases Young Women’s Programming Ability Beliefs

**DOI:** 10.1007/s41979-023-00094-w

**Published:** 2023-05-04

**Authors:** Una Tellhed, Fredrik Björklund, Kalle Kallio Strand, Karolin Schöttelndreier

**Affiliations:** Department of Psychology, Lund University, Lund, Sweden

**Keywords:** Programming, Science center, Intervention, Ability beliefs, Interest, Gender differences

## Abstract

To increase engagement with science and technology, young people around the world are encouraged to attend activities at science centers. But how effective are these activities? Since women have weaker ability beliefs and interest in technology than men, it is especially important to learn how science center visits affect them. In this study, we tested if programming exercises offered to middle school students by a Swedish science center would increase ability beliefs and interest in programming. Students in grades 8 and 9 (*n* = 506) completed a survey before and after visiting the science center, and their ratings were compared to a wait-list control group (*n* = 169). The students participated in block-based, text-based, and robot programming exercises developed by the science center. The results showed that programming ability beliefs increased for women, but not men, and that interest in programming decreased for men, but not women. The effects persisted at a follow-up (2–3 months). The young men reported stronger ability beliefs and interest than the young women at all timepoints. The results imply that science center activities can make programming feel less hard, but adaptations may be needed to also increase interest.

**Supplementary Information:**

The online version contains supplementary material available at 10.1007/s41979-023-00094-w.

## Introduction

In many countries, the labor market is becoming increasingly digitalized (Organization for Economic Co-operation and Development [OECD], 2019; Swedish IT and Telecom Industries, 2021). The tech industry^1^ is growing into an important basis for the economy, and policymakers have realized that to meet future recruitment needs, interest in digital skills such as computer programming must increase in younger generations (Nordic Council of Ministers, 2021; Swedish Agency for Economic and Regional Growth, 2020). This is an important reason why so many science centers have been established around the world (Asia Pacific Network of Science and Technology, 2022; Association of Science and Technology Centers, 2022; European Network of Science Centers & Museums, 2022; Svenska Science Centers, 2022). However, science center activities are rarely evaluated based on how they affect interest in science and technology (Nordic Council of Ministers, 2021; Shoffner & Dockery, 2015; Valla & Williams, 2012). Evaluation is important because it shows whether the current activities meet their aims or require adjustment. In this study, we evaluate how programming activities offered at a Swedish science center impact middle school students’ ability beliefs and interest in programming. We also test for gender differences. There is a particular interest in what increases young women’s tech-related ability beliefs and interest since they tend to be weaker than young men’s (Eccles & Wang, 2016; Hackett, 1995; Lent & Brown, 2019; Master & Meltzoff, 2020; Nordic Council of Ministers, 2021; Rottinghaus et al., 2003; Su & Rounds, 2015; Tellhed et al., 2017, 2018; Watt, 2010; Zell et al., 2015). Very few women enroll in tech-focused educational programs such as computer science, and the tech industry is very strongly dominated by men (European Institute for Gender Equality [EIGE], 2017; Master & Meltzoff, 2020; National Science Foundation, 2021; Nordic Council of Ministers, 2021; Swedish Higher Education Authority, 2020). Attracting more women to the tech industry would help meet its future recruitment needs, and large economic benefits have been estimated from recruiting more women to the STEM sector (Science, Technology, Engineering and Mathematics; EIGE, 2017). Addressing this issue is also important from a gender-equality perspective. People should benefit from, and contribute to, the technological development, regardless of gender (Nordic Council of Ministers, 2021; United Nations Educational, Scientific and Cultural Organization [UNESCO], 2017). It is also concerning that societal gender stereotypes that associate technology with men appear to currently hinder women to develop interest in technology (e.g., Master & Meltzoff, 2020; Nordic Council of Ministers, 2021; Tellhed et al., 2023). The focus of this study is to investigate how visiting a science center may impact middle school students’ ability beliefs and interest in programming. We will next describe these factors and why we selected them as dependent variables.

## Ability Beliefs and Interest

Career choice is affected by a multitude of factors (see Eccles & Wigfield, 2020; Lent & Brown, 2019 for reviews), and some studies have shown that “interest” may be the strongest predictor of career choice (Maltese & Tai, 2011; Rundgren, et al., 2019); that is, if you find programming interesting (or fun), you are more likely to choose to study programming in the future. The centrality of interest for career choice corroborates the well-established social cognitive career theory (SCCT; Lent et al., 1994) and the newer stereotypes, motivation, and outcomes model (STEMO; Master & Meltzoff, 2020). Relatedly, Eccles’s well-known expectancy value theory (EEVT; Eccles, 1987, 1994), which was recently updated to the situated expectancy value theory (SEVT; Eccles & Wigfield, 2020), relates career choice to “values.” In this paradigm, “intrinsic value” (e.g., fun) is similar to the concept “interest” (Eccles & Wigfield, 2020).

If interest is key for career choice, then what shapes our career interests? Drawing on the aforementioned theories, what we believe we are good at, and what careers we believe we would succeed in, is one important basis. There is strong empirical support for this link (e.g., Eccles & Wigfield, 2020; Hackett, 1995; Lent & Brown, 2019; Watt, 2010). In fact, research has shown that *believing* that you have what it takes to do well in a career is more important for developing interest in it than your actual ability, as measured by grades or ability testing (Bandura, 1997; Brown et al., 2008; Lent & Brown, 2019).

The terminology for this type of career choice predictor varies in the literature. Examples are “self-efficacy” (e.g., “I could successfully complete a programming course,” Bandura, 1977, 1997; Lent et al., 1994), “expectancies for success” (Eccles, 1994; Eccles & Wigfield, 2020), and “ability beliefs” (e.g., “I am good at programming,” Master & Meltzoff, 2020). Despite slight differences in definitions, much empirical work has shown a very strong overlap of these constructs (see Eccles & Wigfield, 2020, for a review). We draw on STEMO in this study and use “ability beliefs” as an umbrella term that encompasses both the beliefs that one is “good at programming” and “expects to do well” at programming tasks.

Moreover, a large body of research has shown that gender differences in STEM-related interest are largely explained (statistically mediated) by gender differences in ability beliefs (Bandura, 1977, 1997; Eccles & Wang, 2016; Hackett, 1995; Lent & Brown, 2019; Master et al, 2017; Master & Meltzoff, 2020; Nordic Council of Ministers, 2021; Rottinghaus et al., 2003; Tellhed et al., 2017, 2018, 2022a; Watt, 2010). Women generally rate their STEM ability as being lower compared to men, which contrasts ability tests in this domain that tend to show little to no gender difference, or sometimes even results favoring women’s ability (Ceci et al., 2009; Else-Quest et al., 2010; Hyde, 2005; Hyde et al., 2019; Lin, 2016; Tellhed et al., 2017, 2018; Zell et al., 2015).

To summarize, urgent recruitment needs in the tech industry (Nordic Council of Ministers, 2021; OECD, 2019), as well as the realization that gender stereotypes create barriers for women to develop strong ability beliefs and interest in technology (e.g., Eccles & Wigfield, 2020; Master & Meltzoff, 2020), call for societal interventions to address these issues. We will next describe such initiatives and why they need to be evaluated.

## Literature Review

Around the world, efforts are made to increase children’s skills, ability beliefs, and interest in digital technology. For example, several countries have recently introduced mandatory programming in their curricula (Ministry of Education, 2017; Swedish National Agency for Education, 2018; Uzunboylu et al., 2017), and science centers offer educational experiences to children to increase their understanding and engagement in science and technology (Asia Pacific Network of Science and Technology, 2022; Association of Science and Technology Centers, 2022; European Network of Science Centers & Museums, 2022; Svenska Science Centers, 2022). It is important to scientifically evaluate such societal interventions, to determine whether they fulfill their aims; however, such evaluations are rarely conducted (Nordic Council of Ministers, 2021; Shoffner & Dockery, 2015; Valla & Williams, 2012). For this reason, the current study aims to investigate the effects of programming activities offered by a science center on the visitors’ ability beliefs and interest in programming.

Although societal interventions, such as science centers, are rarely evaluated for their effectiveness, there is a growing research literature on the effectiveness of programming interventions per se. A recent meta-analysis showed that programming interventions can be effective in increasing knowledge and skills, especially when featuring activities that include elements of visualization (e.g., block programming with Scratch) or robots (Scherer et al., 2020). However, very little research has tested the effects of programming interventions on ability beliefs and interest in programming, which as we have explained, should be important predictors of career choice in this domain. Also, gender differences have seldom been reported in the previous work, and since the tech industry is male-dominated, it is important to learn if interventions increase young women’s ability beliefs and interest in programming. The scant literature which has tested the effects of programming interventions on ability beliefs and interest, while also reporting gender differences, has shown that boys and young men tend to report stronger ability beliefs and interest in programming, as compared to girls and young women (Allaire-Duquette et al., 2022; Master et al., 2017; Tellhed et al., 2022). Notably, among college students that had already chosen to study programming or computer science in Taiwan, there was no gender difference in programming ability beliefs (Lin, 2016).

Previous interventions designed to raise ability beliefs and interest in programming have showed mostly promising—but also some surprising—results. A Canadian study recently found that a programming workshop at a science museum eliminated a previous gender difference in programming ability beliefs in a sample aged 10 to 14 (Allaire-Duquette et al., 2022). Similarly, a study in Sweden showed that being taught programming in school increased 12-year-olds’ programming ability beliefs, but surprisingly *reduced* their interest in programming (Tellhed et al., 2022). A study in the USA showed that programming robots in a laboratory increased 6-year-old girls’ ability beliefs and interest in programming, but had no effect on boys (Master et al., 2017). As a contrast, a study in the Netherlands found no effect of a Lego-programming intervention on ability beliefs, but they did not measure specifically *programming* ability beliefs and did not report gender differences (Fanchamps et al., 2021). Lastly, while there are also some qualitative studies suggesting that children tend to find programming activities fun, these studies have not investigated potential gender differences (Fessakis et al., 2013; Kalelioğlu & Gülbahar, 2014; Keren & Fridin, 2014; Rogozhkina & Kushnirenko, 2011).

## Aims and Hypotheses

In this study, we evaluate the effect of a half-day filled with programming activities at a science center on middle school students’ (aged 13–15) ability beliefs and interest in programming. The science center is located in the south of Sweden and offers a variety of activities to middle school classes in the region, including an exhibition on climate change and the focus of this study: programming activities. Visitors to the science center get to try out robot programming, text-based programming, and take part in a lecture that creatively uses dance moves to illustrate the basic elements of programming (see the Methods section for details).

We investigate the students’ ability beliefs and interest in programming prior to their visit at the science center, immediately after the visit, and at a follow-up 2–3 months later, to explore if any intervention effects persist. We also compare the intervention group’s ratings to a “wait-list control group” that gets to visit the science center after data collection is finished. Finally, we also test for gender differences. Based on previous findings (Tellhed et al., 2022), we expect the young women to have weaker interest in programming compared to the young men at the pre-measure, and that a gender difference in programming ability beliefs explains (statistically mediates) this difference. We also explore whether gender moderates any potential intervention effects (i.e., if the effect of the intervention is different for young men compared to young women).

Because the participants in this study are students, they will also partake in their respective schools’ mandatory programming education, which was recently implemented in Sweden (Ministry of Education, 2017; Swedish National Agency for Education, 2018). The participants in the control group will only take part in their school’s programming activities. Thus, we will be evaluating if the science center visit has a positive impact on ability beliefs and interest in programming, in addition to any effect the schools’ education may have. Recall that a previous study showed that programming education in Swedish schools increased students’ ability beliefs but reduced their interest in programming (Tellhed et al., 2022). We expect that the science center’s programming activities will give the participants an extra boost in ability beliefs and test if it counteracts a negative effect that the school’s education may have on interest.

The study’s hypotheses are:

Students report (a) stronger ability beliefs and (b) stronger interest in programming after a visit to a science center, compared to a wait-list control group.
(a) Young men report stronger interest in programming than young women, which (b) is statistically mediated by a gender difference in programming ability beliefs.

We also explore if participant gender moderates any intervention effects and if changes in ability beliefs and interest in programming remain at a follow-up measure, as compared to the pre-intervention measure.

## Method

### Project Description

The data presented here is part of a larger project investigating programming ability beliefs, funded by the Swedish Research Council for Health, Working Life and Welfare (grant # 2019–00334) and by Lund University Faculty of Engineering (grant # P2020-2024).

### Study Design

The study had a quasi-experimental design: The participating school classes were assigned to either an experiment group (the science center intervention group) or a wait-list control group. The latter visited the science center after the data collection was finished. The assignment to condition was not fully randomized, but attempts were made for matching, such that when a school had many classes, some were randomly assigned to the intervention group and some to the wait-list control group.

The data was collected at three different timepoints. The first (T1) was 1–2 months before the visit to the science center. The second (T2) was immediately after the science center activities. The third (T3) was a post-measure taken 2–3 months after the visit to the science center, to test whether any effect of the intervention remained. Due to the COVID-19 pandemic, the science center closed for a period of time, when only a part of the data collection was finished. We therefore repeated the first data collection (T1) for the participating school classes who had finished the T1 survey before the science center closed but had not yet visited the science center. For this subset of participants, the analysis was conducted based on the second collection of the T1 data. However, the same pattern of results was observed for this T1 measure before and after the pandemic restrictions.

### Participants

A total of 715 students in Grade 8 and Grade 9 (age range 13–15) from 11 different schools in the south of Sweden were recruited. We excluded 40 students who either did not complete the first survey, entered the wrong code, or did not provide demographic information. Thus, 675 students were included in the analysis (intervention group; *n* = 506, wait-list control; *n* = 169). Of these students, 578 participated at T2 and 549 participated at T3. The vast majority (96.8%) self-identified with their legal gender (which in Sweden is woman or man) which we used for gender difference analyses. The gender balance was even, with 48.0% young men and 52.0% young women.

### The Programming Exercises

The science center is called Vattenhallen Science Center and is located in the south of Sweden (https://www.vattenhallen.lu.se/english/). It is predominately funded by the Faculty of Engineering at Lund University and is also sponsored by local businesses. It provides different types of science exhibitions, mostly aimed at children and middle school students. The activity we evaluated was a half-day filled with programming exercises, which middle school classes in the larger surrounding region are invited to visit. During their visit to the science center, the school students participated in three types of programming exercises: block-based programming, robot programming, and text-based programming, all which we describe below. Prior to these activities, the students were given an introductory lecture. The researchers that evaluated the activities were not involved in designing them.

#### The Lecture: Unplugged Coding

In the lecture, an educator employed by the science center used “dance moves” to illustrate the basics of programming in a playful and “unplugged” manner. The educator also let robots with sensor technology follow a path on the floor while briefly explaining the technology and the general usefulness of robots, for example, in the labor market. In the “dance programming,” the educator illustrated four basic principles of programming (i.e., sequence, alternative, repetition, and abstraction) using bodily movements to entertain the class. The educator performed sequences of “dance moves” (e.g., “jump,” “turn,” “wave”) to physically act out the principles of programming; they further explained how using the command “repeat” saves time, how “alternatives” (if, else) add complexity to the code, and how “abstraction” further speeds the computer processing by naming a larger sequence. The class was then encouraged to participate in the dance to learn the principles in a playful manner.

After this introduction, the class was divided into three groups that rotated through the three programming stations described next. Staff members (both men and women) helped and encouraged the students at the three stations and answered questions.

#### Block-Based Programming: Microbit

**Fig. 1:**
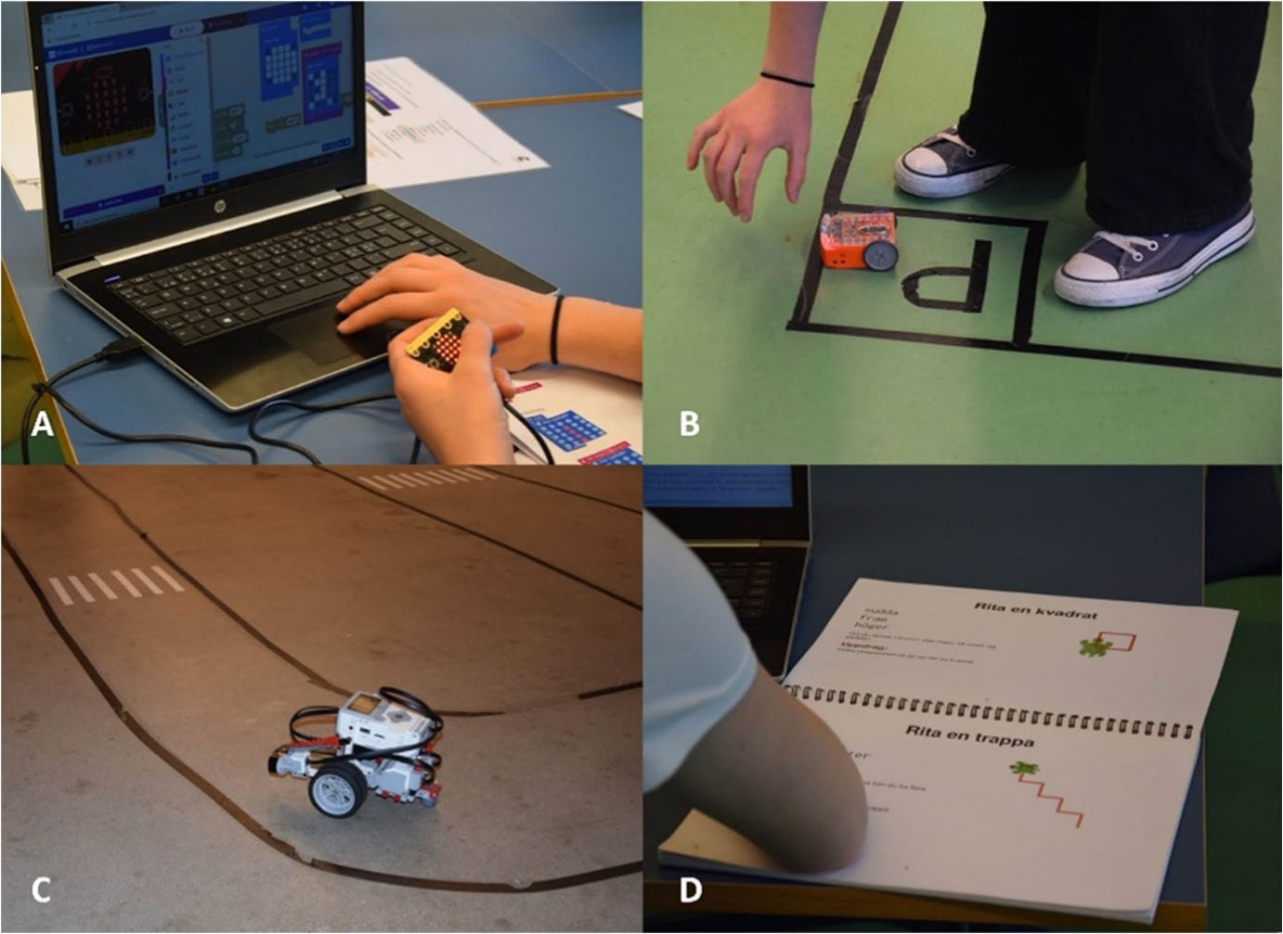
Science center activities at the three programming stations. *Note*. Photo **A** displays Microbit block programming, **B** an Edison robot, **C** a Lego Mindstorms robot, and **D** the text-based programming Kojo

The block-based programming exercise (see Fig. 1A) used Microbit Technology, which is a small computer with 25 pixels (LED lights) that can be turned on and off with block program instructions (https://microbit.org/). Block programming is a visual programming language which is easier to learn than text-based programming, and that has been shown to have positive effects on learning (Scherer et al., 2020). The students followed an instruction sheet with directions, including how to code the Microbit to resemble a traffic light.

#### Robot Programming: Edison and Lego Mindstorms

The robot programming exercise also applied block-based programming. The participants either instructed an Edison Robot (https://meetedison.com/; see Fig. 1B) or a Lego Mindstorms robot (https://www.lego.com/en-us/themes/mindstorms; see Fig. 1C) to follow a U-shaped racing track on the floor. The principles for programming these robots are quite similar, and the students used blocks to instruct the robot to drive forward and turn. The commands were repeated until they made the robot attain the angle and speed just right to pass the track.

#### Text-Based Programming: Kojo

The text-based programming exercise is called Kojo (see Fig. 1D) and is based on the programming language Scala (https://docs.kogics.net/ikojo.html). In the exercise, the students wrote code (e.g., “repeat (4) {forward; right}”) to make a turtle move on the computer screen to draw a figure (e.g., a square). A booklet next to the computer described the commands related to the four basic principles of programming that the students had practiced with dance moves in the introductory lecture (i.e., sequence, alternative, repetition, abstraction).

### Measures and Procedure

The data was collected during school hours through the survey tool Qualtrics. All material was in Swedish. The measures presented in this article represent part of the larger project (see Project Description) and other results will be presented elsewhere. The dataset, code, and the items measuring programming ability beliefs can be found in the open science data repository (https://osf.io/u69n4/). Each student received an ice cream gift card after having filled out the T3 survey.

#### Programming Ability Beliefs

Programming ability beliefs were measured with a 7-item scale (e.g., “I am good at programming,” “I have confidence in my ability to program”) adapted from Kong et al.’s (2018) scale but revised to also include two reversed items (inspired by Tek et al., 2018). It had a 5-point response scale, ranging from 1 (“strongly disagree”) to 5 (“strongly agree”). The scale has revealed satisfactory convergent and content validity (Kong et al., 2018). Cronbach’s alpha was .82 at T1, .85 at T2, and .81 at T3.

#### Programming Interest

Interest in programming was measured with the item “How interesting do you think programming is?,” which was rated on a 5-point scale ranging from 1 (“not at all interesting”) to 5 (“extremely interesting”). This item was adapted from the well-established SCCT framework and has previously been validated (Lent & Brown, 2006).

#### Perceptions After the Visit

For descriptive purposes, the participants were also asked at T3 to reflect on the science center visit and estimate how it affected them based on the following questions: “How much did you feel you learned from the robot programming at [the science center]?,” “Did the visit make you curious to learn more about programming?,” “Have you talked to friends about the visit to [the science center] after you were there?,” and “Have you talked to your family about the visit to [the science center] after you were there?” Responses were made on a 5-point scale ranging from 1 (“nothing at all”) to 5 (“very much”). For the last two items, the Spearman-Brown coefficient was .77, so an index was created by calculating the mean for the students’ communications about the visit.

### Data Analysis

The data was analyzed in R version 4.1.2 (R Core Team, 2021). All categorical variables used in the analyses were coded using Helmert contrasts (e.g., gender coded as −1 for young men, 1 for young women). Hypothesis testing for the longitudinal data was carried out using linear mixed-effects modeling. We fit linear mixed-effects models using the lme4 package (Bates et al., 2015) with *p*-values generated using the lmerTest package (Kuznetsova et al., 2017). Interaction effects were interpreted by comparing estimated marginal means using the emmeans package (Lenth, 2022). All models were tested to ensure that there were no violations of the assumptions of linear mixed-effects modeling, namely normality, linearity, multicollinearity, homoscedasticity, and influential outliers.

## Results

Table 1 presents descriptive statistics for each variable across the three timepoints. Preliminary analyses were conducted to confirm that there were no significant differences between the intervention and control group in the outcome variables at T1. Overall, the descriptive statistics illustrate that the students who visited the science center had learned a moderate amount (i.e., mean ratings close to the midpoint of the scale) and had become somewhat more curious about programming. They had also talked a little with friends and family about the science center visit.

**Table 1:** Descriptive statistics across the three data collection timepoints

	Intervention group						Control group					
	Total		Women		Men		Total		Women		Men	
Variable	*n*	*M* (*SD*)	*n*	*M* (*SD*)	*n*	*M* (*SD*)	*n*	*M* (*SD*)	*n*	*M* (*SD*)	*n*	*M* (*SD*)
Ability beliefs												
Timepoint 1	506	2.77 (0.82)	261	2.55 (0.77)	245	3.00 (0.80)	169	2.81 (0.69)	90	2.61 (0.66)	79	3.04 (0.65)
Timepoint 2	429	2.99 (0.83)	222	2.77 (0.77)	207	3.21 (0.83)	149	2.88 (0.67)	80	2.71 (0.68)	69	3.08 (0.62)
Timepoint 3	401	2.89 (0.81)	209	2.71 (0.77)	192	3.09 (0.81)	148	2.93 (0.69)	79	2.79 (0.68)	69	3.08 (0.68)
Interest												
Timepoint 1	506	2.80 (1.21)	261	2.35 (1.08)	245	3.28 (1.17)	169	2.86 (1.13)	90	2.47 (1.07)	79	3.32 (1.03)
Timepoint 2	429	2.75 (1.15)	222	2.46 (1.08)	207	3.06 (1.13)	149	2.57 (1.07)	80	2.25 (0.92)	69	2.94 (1.11)
Timepoint 3	401	2.70 (1.13)	209	2.38 (0.99)	192	3.05 (1.16)	148	2.58 (1.03)	79	2.23 (0.97)	69	2.99 (0.95)
Post-visit measures (T3)												
Felt I learned	401	2.78 (0.99)	209	2.82 (0.91)	192	2.72 (1.07)						
Became curious	401	2.44 (1.09)	209	2.26 (1.01)	192	2.63 (1.14)						
Talked about the visit	401	2.13 (0.95)	209	2.17 (1.00)	192	2.09 (0.89)						

*Note*. Total = both women and men. T3 = Timepoint 3

### Hypothesis 1a: Changes in Ability Beliefs Over Time

To test Hypothesis 1a, i.e., whether the science center visit influenced programming ability beliefs, we created a linear mixed-effects model (Model 1) with ability beliefs as the outcome variable and linear time, quadratic time, condition (intervention, control), and gender as predictor variables. We included an interaction between condition, gender, and time for both linear and quadratic time, to investigate whether the over-time change in ability beliefs would be contingent on condition as well as to explore whether this effect would be moderated by gender. Lastly, we included a random intercept and slope for each student as well as a random intercept for each school. The results for Model 1 are presented in Table 2. There was a significant effect of gender in that the young men reported stronger ability beliefs at T1 than did the young women. Across the three timepoints, there was an overall linear increase in ability beliefs across both gender and condition. However, a significant interaction effect between gender and linear time revealed that the linear increase in ability beliefs was only found for young women (*b* = 0.07, 95% CI [0.04, 0.10]), not for young men (*b* = 0.02, 95% CI [−0.02, 0.05]). Thus, Hypothesis 1a was supported, but only for the young women. Although young women showed an increase in programming ability beliefs, an independent samples *t*-test revealed that the gender difference found at T1 remained significant at T3, *t*(537.44) = 5.49, *p* < .001, *d* = 0.47.

**Table 2:** Summary statistics for Model 1 predicting programming ability beliefs

Predictor	*β*	*p*	95% CI	*R*^2^
Time_Linear_	0.06	< .001	[0.03, 0.09]	.003
Time_Quadratic_	−0.04	.005	[−0.07, −0.01]	.001
Gender (base = young men)	−0.24	< .001	[−0.31, −0.16]	.015
Condition (base = intervention)	0.00	.407	[−0.12, 0.12]	.002
Gender × Time_Linear_	0.04	.021	[0.01, 0.07]	.001
Gender × Time_Quadratic_	0.00	.609	[−0.02, 0.04]	< .001
Condition × Time_Linear_	0.00	.890	[−0.03, 0.03]	< .001
Condition × Time_Quadratic_	0.04	.012	[0.01, 0.06]	.001
Gender × Condition	0.03	.515	[−0.05, 0.10]	< .001
Gender × Condition × Time_Linear_	0.01	.347	[−0.02, 0.04]	< .001
Gender × Condition × Time_Quadratic_	0.00	.990	[−0.03, 0.03]	< .001

*Note*. Model *R*_*m*_^2^ = .072, *R*_*c*_^2^ = .727

**Fig. 2:**
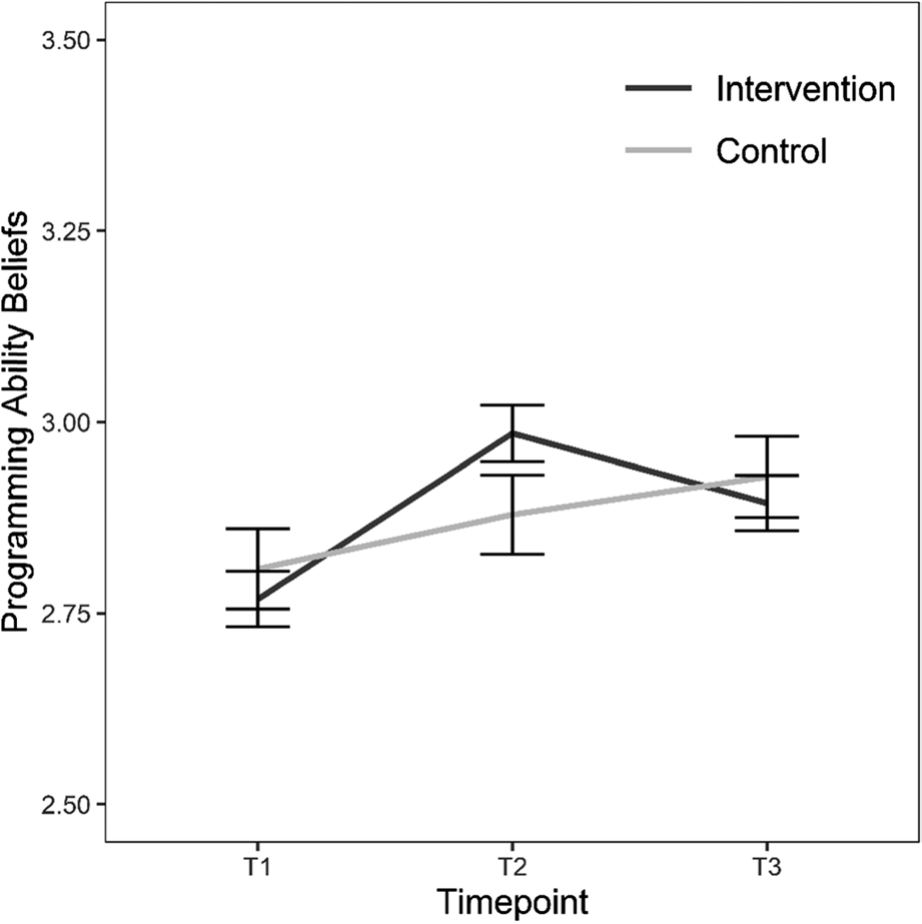
Quadratic Time × Condition interaction for programming ability beliefs in Model 1 (with standard error bars)

Notably, there was also a significant interaction effect between condition and quadratic time, which is illustrated in Fig. 2. The estimated marginal means revealed that only the intervention group showed a quadratic over-time change in ability beliefs, which was marked by an increase between T1 and T2 followed by a slight decline between T2 and T3. The T3 measure for the intervention group was higher than their T1 measure, *t*(400) = 3.96, *p* < .001, *d* = 0.15. No curvilinear trend was found for the control group over and beyond the linear trend. There was no significant interaction effect between condition and linear time, nor between gender and quadratic time. Lastly, there was no significant three-way interaction in Model 1.

### Hypothesis 1b: Changes in Interest Over Time

Next, we investigated whether the science center visit influenced the students’ interest in programming (Hypothesis 1b), as well as explored the moderating effect of gender, by means of linear mixed-effects modeling. This model, Model 2, contained the same fixed and random effects as Model 1 but with interest as the outcome variable. The results for Model 2 are presented in Table 3. As in Model 1, there was a significant effect of gender in Model 2, by which young women were less interested in programming than were young men at T1. There was also a significantly negative linear main effect of time across both gender and condition. Notably, this linear time effect was superseded by two interaction effects. First, there was a significant Condition × Linear Time interaction, by which students in the intervention group showed a less pronounced decline in interest in programming (*b* =  −0.05, 95% CI [−0.08, −0.01]) compared to the control group (*b* =  −0.13, 95% CI [−0.19, −0.07]). Because both groups showed a decline in interest over time, we did not find support for Hypothesis 1b. Second, there was a significant Gender × Linear Time interaction, by which young men’s interest declined over time (*b* =  −0.13, 95% CI [−0.18, −0.08]), whereas young women’s interest did not (*b* =  −0.05, 95% CI [−0.09, 0.00]). As in Model 1, the gender difference in interest found at T1 in Model 2 remained significant at T3, *t*(523.35) = 7.71, *p* < .001, *d* = 0.66.

**Table 3:** Summary statistics for Model 2 predicting interest in programming

Predictor	*β*	*p*	95% CI	*R*^2^
Time_Linear_	−0.08	< .001	[−0.11, −0.04]	.005
Time_Quadratic_	0.03	.050	[0.00, 0.06]	.001
Gender (base = young men)	−0.32	< .001	[−0.39, −0.25]	.019
Condition (base = intervention)	−0.04	.264	[−0.18, 0.09]	.002
Gender × Time_Linear_	0.04	.025	[< 0.01, 0.07]	.001
Gender × Time_Quadratic_	−0.04	.022	[−0.06, −0.01]	.001
Condition × Time_Linear_	−0.04	.025	[−0.07, < −0.01]	.001
Condition × Time_Quadratic_	0.02	.119	[−0.01, 0.05]	< .001
Gender × Condition	−0.01	.754	[−0.08, 0.06]	< .001
Gender × Condition × Time_Linear_	−0.02	.329	[−0.05, 0.02]	< .001
Gender × Condition × Time_Quadratic_	0.00	.834	[−0.03, 0.03]	< .001

*Note*. Model *R*_*m*_^2^ = .112, *R*_*c*_^2^ = .701

For quadratic time, there was a marginal albeit nonsignificant main effect. However, there was a significant interaction effect between gender and quadratic time. The results revealed that for the young men, the linear decrease in time was superseded by a curvilinear pattern. The pattern showed that young men’s interest in programming decreased from T1 to T2 but was unchanged between T2 and T3, across both the intervention group and the control group. No quadratic time effect was found for the young women, nor was there any interaction effect between quadratic time and condition. Lastly, there were no significant three-way interaction in Model 2.

### Hypothesis 2: Mediation Analyses of Gender Differences in Interest

In Model 1, we found that young men reported stronger ability beliefs for programming compared to young women across all three timepoints. Similarly, Model 2 illustrated that there was a gender difference concerning interest in programming: across all three timepoints, young men reported a stronger interest in programming than did young women, which supported Hypothesis 2a. Furthermore, ability beliefs and interest in programming were significantly correlated at each of the three timepoints in the full sample (including both young men and women), *r*_S_ = .53–.60. These findings allowed us to test whether the gender difference in programming interest could be explained by a gender difference in programming ability beliefs (Hypothesis 2b). We used the lavaan package (Rosseel, 2012) to conduct a mediation analysis by means of structural equation modeling, using data from T1. A direct path was added between gender and interest in programming, as well as an indirect path via ability beliefs.

**Fig. 3:**
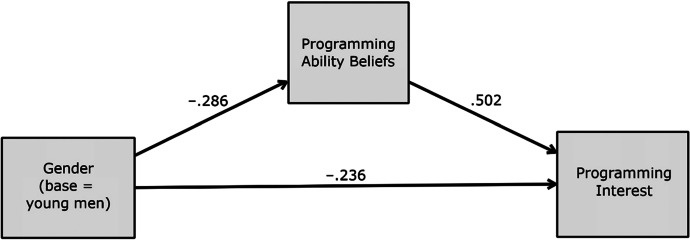
Mediation analysis of gender differences in interest in programming via ability beliefs at T1 (standardized path coefficients)

The structural model with standardized coefficients is illustrated in Fig. 3 (see Table A1 in the Supplemental Material for all parameter estimates). As in Model 2, there was a significant direct effect of gender on interest in programming (*p* < .001), explaining 5.6% of the variance in interest, by which young women were less interested in programming than were young men. There was also a significant indirect effect of gender via ability beliefs (*p* < .001), explaining 2.1% of the variance in interest. This indirect effect constituted 37.9% of the total effect of gender on interest in programming and supported Hypothesis 2b by identifying ability beliefs as a partial mediator of the relationship between gender and interest in programming. Additional analyses revealed that the partial mediational effect was consistent across the three timepoints, which further aligned with our predictions.

## Discussion

This study investigated if programming activities at a science center could increase middle school students’ ability beliefs and interest in programming, as well as tested for gender differences in these effects. We will first discuss the science center visit’s effect on programming ability beliefs, followed by the effects on interest in programming.

### Effects on Programming Ability Beliefs

The results showed that programming ability beliefs increased for students who visited the science center, both compared to a pre-measure and to a wait-list control group, which supported our hypothesis. Encouragingly, the effect on ability beliefs also persisted at a follow-up measure 2–3 months after the science center visit, although the effect size was small. Interestingly, when exploring potential gender differences in the intervention effect, the results showed that it was only the young women’s ability beliefs that increased after taking part in the science center activities—the young men’s ability beliefs were not affected. This finding matches a previous study in the USA, where a programming intervention with younger children similarly only increased ability beliefs in girls, not boys (Master et al., 2017).

We cannot determine what aspects of the intervention impacted the young women’s ability beliefs, because we tested the overall effects of the science center visit. However, according to social cognitive theory (Bandura, 1977, 1997), trying out programming and finding that you can master it (mastery experience), being encouraged by the staff at the science center (social persuasion), who also act as role models (vicarious experience), and programming in a low-stress environment (arousal) may all have contributed to the effect.

Even though young women’s ability beliefs were increased by the visit at the science center, a gender difference in ability beliefs remained. This result differed from two previous studies that found no significant gender difference in programming ability beliefs after a programming intervention (Allaire-Duquette et al., 2022; Master et al., 2017). This difference across studies may possibly be related to age. The participants in the current study were older (aged 13–15) than in the previous studies, which employed children at the age of 6 (Master et al., 2017) and 10–14 (Allaire-Duquette et al., 2022) as participants. Previous research suggests that a critical period to impact science interest may be around the ages of 8–13 (Dou, 2019; Master et al., 2017; Master & Meltzoff, 2020; Shoffner & Dockery, 2015).

Increasing young people’s programming ability beliefs is important since it is very strongly related to the formation of career interest (e.g., Eccles, 1987, 1994; Eccles & Wigfield, 2020; Lent & Brown, 2019; Lent et al., 1994; Master & Meltzoff, 2020), which may be the most important predictor of career choice (Maltese & Tai, 2011; Rundgren et al., 2019). An important aim of this study was therefore to investigate if the science center activities could increase the students’ interest in programming. This is not obvious, given that a recent study showed that programming education in school *reduced* interest in programming, despite increasing programming ability beliefs (Tellhed et al., 2022).

### Effects on Programming Interest

The results showed that interest in programming decreased continuously over all three timepoints in this study. However, the decrease was larger in the control group, as compared to the group that visited the science center. This means that the participants who only took part in their schools’ mandatory programming education became less interested in programming, which corroborated results from a previous study (Tellhed et al., 2022). Encouragingly, although the intervention group also showed a decline in interest over time, the science center visit appeared to inhibit this decrease to some degree.

Notably, the decrease in interest was only found in young men. As a contrast, the young women’s interest in programming did not decrease over time, although it was weaker than the young men’s interest at all timepoints. This finding can be related to the study on younger children in the USA (Master et al., 2017), where programming a robot increased only girls’ interest in programming. It is also consistent with a study in which women in STEM educations at a university level rated school excursions in their childhood as more important for their interest development in STEM, than did men in STEM educations (Rundgren et al., 2019). The reason why the young women’s interest in programming persisted in our study (albeit at a low level) could relate to their increased programming ability beliefs over time. In line with previous research, and corroborating SCCT, EEVT, SECT, and STEMO, ability beliefs were quite strongly related to interest in this study and partially mediated the gender difference in interest^2^ (Eccles, 1987, 1994; Eccles & Wigfield, 2020; Lent & Brown, 2019; Lent et al., 1994; Master & Meltzoff, 2020).

## Limitations

There are several limitations to the present study. The external validity is limited because we only investigated the programming activities of a single science center in Sweden, and for one age group (ages 13–15). We encourage further scientific evaluations of societal programming interventions, and cross-country comparisons, to test whether effects vary or are similar for different types of interventions, in different parts of the world, and for different age groups.

The study had an unusually strong research design as compared to standard evaluations of societal interventions: It had three repeated measures, a large participant sample and two conditions (intervention vs. control). However, the internal validity was limited since we could not randomize the visiting school classes to conditions, which meant the design was quasi-experimental. However, we matched the assignment to conditions, such that when a school sent more classes than one to the science center, some were assigned to the wait-list control group and some to the intervention group. We tested for potential differences between conditions at the pre-measure, and none was significant. Furthermore, the results of the control group resembled the results of a previous study, which investigated development over time in ability beliefs and interest in programming in a similar age group (Tellhed et al., 2022). However, we recommend future studies to replicate the present study while also attempting to implement individualized randomization. Future studies may also want to test if variation in participant factors, such as school grades or programming test performance, moderate the impact of programming interventions. Moreover, and as previously mentioned, we investigated the effects of the intervention as a whole and can therefore not determine if the different components of the intervention (e.g., the different programming activities, encouragement from staff, the role model exposure) impacted ability beliefs and interest to different degrees. We encourage replications which disentangle the impact of different aspects of STEM interventions.

This study had a specific focus on ability beliefs in relation to programming, where we employed a carefully adapted scale. The programming interest measure was limited to just one item, and we encourage future replications to expand this measure to include more aspects such as intrinsic value measures from the EEVT paradigm. Lastly, the results revealed no significant three-way interactions (Condition × Gender × Timepoint). This could reflect that no such interactions exist or that they were undetected due to a lack of power; weak three-way interaction effects would require an even larger participants sample than we had in this study.

## Implications

This study showed that a single visit to a science center increased young women’s (but not young men’s) programming ability beliefs. The visit also counteracted the negative effect that mandatory programming education in school had on interest in programming, such that young women’s interest did not decline over time. The positive effects of the visit remained at a follow-up measure 2–3 months later. Understanding that a single science center visit can have these encouraging effects is important since it suggests that societal interventions like this may be effective and worth investing in.

It may be helpful for science centers to collaborate with psychologists to design activities which may have a positive impact on young people’s STEM-related interest. Firstly, age is one important factor to consider. Based on previous research, it is advisable to target children and youth around the age of 8–13, to boost ability beliefs and interest in STEM (Dou, 2019; Master et al., 2017; Master & Meltzoff, 2020; Shoffner & Dockery, 2015). When targeting older youths, which are more set in their interests, efforts should be made to match activities to the age-related preferences of the visitors. Parts of the current setup at the science center evaluated in this study included programming robots and performing dance moves to play out coding sequences. Based on the finding that the young men’s interest in programming decreased after the visit, it is possible that the activities fit younger children better. To generate ideas on intervention that are more appropriate for certain age groups, science centers could use reference groups with a matching age range to that of their visitors.

Secondly, it is important to consider gender differences when developing STEM interventions aimed to increase interest. The results of this study corroborated a large body of previous research that has found a gender difference in interest in STEM, which is partially explained by girls’ and women’s typically weaker ability beliefs (e.g., Eccles & Wigfield, 2020; Hackett, 1995; Lent & Brown, 2019; Watt, 2010). Previous research suggests that men may develop ability beliefs in STEM mainly through mastery experience (trying something out), while encouragement and female role models are key for women’s ability beliefs development (Zeldin et al., 2008). The science center we evaluated employed both men and women as instructors, which may have contributed to the positive effect that the visit had on young women. To further boost young women’s interest in technology, science centers may also want to consider stressing how technology can be used to help people and benefit society, as women tend to have stronger such “communal” career goals (e.g., Brown et al., 2015; Diekman et al., 2011, 2016; Rundgren et al., 2019; Shoffner & Dockery, 2015; Tellhed et al., 2018).

Social psychological theory ultimately relates gender differences in ability beliefs and interest in technology to gender stereotypes associating technology more with men (see Master et al., 2020, for a review). To reduce barriers preventing girls and women from developing similar levels of tech interest to men, efforts are needed to counteract stereotypes that portray men as better at technology than women (e.g., Master & Meltzoff, 2020; Tellhed et al., 2023). We suggest that efforts should be taken to spread facts about “gender similarity”^3^ in STEM abilities, to counteract current gender stereotypes (Hyde et al., 2019; Liu et al., 2021; Zell et al., 2015). Also, schoolteachers should be informed that gender differences in STEM ability tend to be very small since previous research has shown that teachers in Sweden tend to associate technology strongly with men (Tellhed et al., 2023). Awareness of one’s own gender stereotypes can counteract the likelihood of unintentionally conveying them to students, particularly when motivated to avoid doing so (Cox & Devine, 2023).

Lastly, science centers may also want to encourage visitors to share their experiences with others. A previous study showed that an important predictor of college students’ STEM identity was how much they had talked about science with friends and family in their childhood (Dou et al., 2019). In this study, the visitors had only spoken about the science center visit to a small degree with friends and family. One idea may be to ask the participating schoolteachers to create assignments where students tell others about the visit (perhaps through a video clip). Science centers may also want to offer discount checks for visitors to come back with their friends and family to encourage more science communication in the community.

## Conclusions

The results of this study suggest that science center activities can have positive effects on middle school students’ ability beliefs and interest in programming. Visiting a science center for half a day particularly boosted young women’s beliefs that they can be good at programming and counteracted the negative effect that school education had on the perception that programming can be fun. Encouragingly, the positive effects remained 2–3 months after the visit, but despite the benefits for young women, the young men still reported stronger ability beliefs and interest in programming than the young women. This suggests that more efforts need to be taken to reduce barriers for women to freely develop interest in the fast-growing tech industry and to make sure that citizens of all genders will take part in the increasingly digitalized society.

The content of the manuscript has not been previously published, or submitted for publication elsewhere. All authors have approved the final manuscript for submission.

## Supplementary Information

Below is the link to the electronic supplementary material.

Supplementary file1 (DOCX 36 KB)

## Footnotes

1 The tech industry includes IT, Telecom and game development, health (healthtech), finance (fintech), and education (*edtech*; Swedish IT & Telecom Industries, 2021).

2 As mentioned in the “Introduction” section, EEVT and SEVT employ the term “intrinsic value” (e.g., fun), which is similar to the concept interest (Eccles & Wigfield, 2020).

3 Janet Shibley Hyde (2005) coined this term for the finding that most psychological gender differences are very small in effect size.
