# Supplementary material for: “Programming Is Not That Hard!” When a Science Center Visit Increases Young Women’s Programming Ability Beliefs
Source: J STEM Educ Res. 2023 May 4:1–23. Online ahead of print. doi: 10.1007/s41979-023-00094-w (PMC10159224; doi:10.1007/s41979-023-00094-w)
Supplement: Supplementary file 1 — Supplementary file1 (DOCX 36 KB) [file 41979_2023_94_MOESM1_ESM.docx]

**Supplemental Material**

**Table A1**

*Standardized Parameter Estimates for the Structural Equation Model (Mediation Analysis)*

| Parameter | Estimate (*SE*) | *p* | 95% CI |
| --- | --- | --- | --- |
| Regressions |  |  |  |
| Gender (base = men) → Ability Beliefs | –.286 (.035) | < .001 | [–.355, –.217] |
| Gender → Programming Interest | –.236 (.033) | < .001 | [–.300, –.173] |
| Ability Beliefs → Programming Interest | .502 (.030) | < .001 | [.443, .561] |
|  |  |  |  |
| Residual Variance |  |  |  |
| Ability Beliefs | .918 (.020) | < .001 | [.879, .957] |
| Programming Interest | .624 (.031) | < .001 | [.564, .684] |

*Note*. Bootstrapping (*n* = 10,000) was used to generate 95% CIs.
